# Database of *Trypanosoma cruzi *repeated genes: 20 000 additional gene variants

**DOI:** 10.1186/1471-2164-8-391

**Published:** 2007-10-26

**Authors:** Erik Arner, Ellen Kindlund, Daniel Nilsson, Fatima Farzana, Marcela Ferella, Martti T Tammi, Björn Andersson

**Affiliations:** 1 Department of Cell and Molecular Biology, Karolinska Institutet, Stockholm, Sweden; 2 Department of Biological Sciences, Biochemistry, National University of Singapore, Singapore

## Abstract

**Background:**

Repeats are present in all genomes, and often have important functions. However, in large genome sequencing projects, many repetitive regions remain uncharacterized. The genome of the protozoan parasite *Trypanosoma cruzi *consists of more than 50% repeats. These repeats include surface molecule genes, and several other gene families. In the *T. cruzi *genome sequencing project, it was clear that not all copies of repetitive genes were present in the assembly, due to collapse of nearly identical repeats. However, at the time of publication of the *T. cruzi *genome, it was not clear to what extent this had occurred.

**Results:**

We have developed a pipeline to estimate the genomic repeat content, where shotgun reads are aligned to the genomic sequence and the gene copy number is estimated using the average shotgun coverage. This method was applied to the genome of *T. cruzi *and copy numbers of all protein coding sequences and pseudogenes were estimated. The 22 640 results were stored in a database available online. 18% of all protein coding sequences and pseudogenes were estimated to exist in 14 or more copies in the *T. cruzi *CL Brener genome. The average coverage of the annotated protein coding sequences and pseudogenes indicate a total gene copy number, including allelic gene variants, of over 40 000.

**Conclusion:**

Our results indicate that the number of protein coding sequences and pseudogenes in the *T. cruzi *genome may be twice the previous estimate. We have constructed a database of the *T. cruzi *gene repeat data that is available as a resource to the community. The main purpose of the database is to enable biologists interested in repeated, unfinished regions to closely examine and resolve these regions themselves using all available shotgun data, instead of having to rely on annotated consensus sequences that often are erroneous and possibly misleading. Five repetitive genes were studied in more detail, in order to illustrate how the database can be used to analyze and extract information about gene repeats with different characteristics in *Trypanosoma cruzi*.

## Background

Large duplications and other long repeats are common features in the genomes of higher organisms, and they often have important biological consequences. During evolution, duplications may give rise to diversified variants of the same protein, and may provide the genome with unexpected combinations and modularity in the formation of more complex genes. Chromosomal rearrangements between paralogous sequences can cause disease [1], and gene copy numbers have been shown to be correlated with mRNA expression levels e.g. [2].

Repetitive DNA is the major cause of misassemblies in shotgun sequencing, and most genome projects labeled as "finished" still have unresolved repeats [3,4]. In the human genome, 3–4% of the euchromatic portion is estimated to consist of duplications, 30–40% of which are likely to have caused misassembly [5,6]. Similarly, almost 60% of the duplicated part of the genome of mouse (approximately 2%) is believed to be misassembled or collapsed in the draft working sequence [7]. Assembly errors include erroneous copy numbers of repeated genes, errors in gene and chromosomal order, as well as inaccuracies in the actual sequence of individual genes due to the mixing of reads from different repeat copies.

Users of draft genome sequences for different organisms need to be aware of these complications. As the draft sequence often is a starting point for experiments, time, effort and resources may go to waste if the target region in reality is different from the sequence retrieved from the database. Confusion of paralogous and allelic SNPs, misdesigned primers and failed cloning are just a few of the problems that could arise from relying on erroneously assembled sequences. Although tools for repeat and misassembly analysis of bacterial genomes have recently been developed [8], genome browsers generally contain little or no information regarding which parts of the assembly have an unusually large number of aligned sequences and therefore may contain collapsed repeats. The recently presented Assembly Archive [9] is an attempt to rectify this issue, and it will hopefully expand and become a useful resource in the future.

*Trypanosoma cruzi *has one of the most repeated genomes known to date. Over 50% of the genome consists of repeated sequences [10]. In addition to being highly repetitive, the reference clone for the sequencing project, CL Brener, is a hybrid. The parasite is diploid, but with a high degree of difference between homologous chromosomes and occasional triploidy [11]. The high degree of repeated sequence makes *T. cruzi *a suitable system for studying different kinds of repeat phenomena, such as gene conversion, chromosome rearrangements, and other evolutionary aspects. Moreover, genes that play a key role in the host-parasite interaction, mostly surface proteins, are often repeated, along with many other types of genes. However, the draft sequence of *T. cruzi *suffers from the problems described above, i.e. collapsed regions and misassemblies due to the repeated nature of the genome. While non-repeated sequences have an accurate representation in the draft sequence, the gene repeats have only been subjected to a shallow, partial analysis and the draft genomic sequence in these regions is often incomplete and misleading. Partial sequencing of one of the parental strains of CL Brener has made it possible to separate and annotate the homologous chromosomes in many parts of the genome. However, many regions are annotated as merged haplotypes and many more are annotated as repetitive and impossible to resolve between the two haplotypes.

An example of misassembly in the *T. cruzi *genome is the genes coding for monoglyceride lipase. It has been annotated once in the genome. A closer look at the alignment depth of reads matching this gene reveals that it is actually present in approximately 50 copies in the *T. cruzi *genome. Another example is trans-sialidase (see below), where twelve out of 1 430 annotations have been identified as having trans-sialidase activity. In reality, a majority of these have a consensus sequence that is not actually supported by shotgun sequence reads upon closer inspection. Although protein translations of annotated genes in *T. cruzi *were subjected to analysis by TRIBEMCL [12] within the WGS project, and annotated genes also were grouped in Clusters of Orthologous Genes (COGs) using Blast, a repeat analysis on shotgun coverage was only performed for a limited number of genes.

A few in-depth studies of repeated coding sequences in *T. cruzi *have been made [13,14]. The regions analyzed consist of relatively short repeat units for which the entire repeat cassette can be compared using ClustalW [15] and similar tools. This approach is not feasible when the repeat cassette is longer than a shotgun read length, which is the case for most of the *T. cruzi *gene repeats. There is clearly a need for a resource that simplifies the task of analyzing the repeated regions in the parasite. Gene copy numbers have in a few cases been determined empirically; see for example [16,17] and [18]. These were laborious studies that were mostly made on other strains of *T. cruzi *than the genome reference strain. A resource listing the genes that exist in multiple copies does not replace such confirming analyses, but provides a starting point for which genes should be analyzed.

We here present a comprehensive, genome-wide analysis of the predicted protein coding regions and pseudogenes in *T. cruzi*, resulting in the discovery of approximately 20 000 new protein coding sequences and pseudogenes. The study does not include non protein-coding genes, such as RNA genes, nor other types of repeats. The results are summarized in a database that is available to the community [19]. The analysis has been performed using all shotgun sequence reads from the *T. cruzi *genome sequencing project, 26% of which never made it into the original assembly due to problems with repeats and interhomolog polymorphisms – and are thus previously un-analyzed. To illustrate the utility of the database, we also provide an in-depth study of five repetitive genes. This highlights different characteristics of the *T. cruzi *gene repeats, such as their organization between homologs and degree of similarity. The analysis was performed using a sequence similarity search software (GRAT [20]) and an assembly analysis and editing tool (DNPTrapper [21]) that were previously developed at our laboratory. These tools, as well as the database itself, are available for public use at the URL cited above. We argue that databases such as the one presented here are essential as complements to existing databases of unfinished genomes, and we suggest that this approach for gene repeat analysis and resolution can be used for other genome projects.

## Methods

### Definitions

Unless otherwise stated, the word *gene *refers to a protein coding sequence or a related pseudogene, while *annotated gene *refers to an open reading frame in the consensus sequence of the *T. cruzi *assembly (project accession AAHK00000000 in GenBank, [10]) that was annotated as representing a *gene*. Given a *gene*, a *gene copy *is defined as another sequence similar to the original one within the parameters used in this paper (see below), and the *copy number *refers to the number of *gene copies *that are estimated to exist for a given *gene*. When discussing a particular *annotated gene*, a *variant *is defined as another *annotated gene *with the same functional classification. A *pseudogene *is a *variant *that has been annotated as having lost its protein coding function, due to, for example, a frame shift. Due to the large difference between the homologous chromosomes, we have, in this study, chosen to treat the two allelic copies of a gene as two gene copies, thereby estimating the majority of genes, the non-repetitive genes, to exist in two copies.

### Multiple alignments

Multiple alignments of annotated genes and shotgun reads matching them were built to assess the copy number of each gene and pseudogene using GRAT. Also, multiple alignments built from annotated genes were constructed to cluster the protein coding sequences and pseudogenes based on sequence similarity. The alignments of annotated genes and shotgun reads were used, in cases of large alignment depth, to assess the borders of a repeat unit. The details of multiple alignment construction are described below. All alignments were made using nucleotide sequences, as were all analyses unless otherwise stated. The shotgun reads were obtained from the three collaborating laboratories in the genome sequencing project: TIGR, SBRI and our own.

### Coding sequences as queries

All annotated protein coding genes and corresponding pseudogenes were used as query sequences to build multiple alignments from a database of all reads from the *T. cruzi *whole genome shotgun project. The tolerated polymorphism rate was set to 5%, and thus sequences more divergent than 95% were separated and those more similar than 95% were grouped together. This setting was chosen based on an estimation of which sequences were separated in the original shotgun assembly. It is highly unlikely that sequences with lower similarity than 95% have collapsed in the assembly. Although there are cases in the assembly where sequences with higher similarity have been separated correctly, the limit was chosen to ensure that all sequences that collapsed in the assembly would be included in this study.

The reads could align to more than one coding sequence. In fact, in the case of a coding sequence and its allele, all reads aligning to a certain gene should, with our 95% similarity setting, also align to its allele. This would result in a coverage corresponding to two copies for both alleles. Note that this is different from how the total gene count was calculated, where every read was only counted once.

The multiple alignments were analyzed for alignment depth. The standard deviation of a sampling of the depth every 100 bp was calculated. The copy numbers of the individual annotated genes were calculated based on the average depth in the alignment and an average coverage of 7 in single copy regions from the shotgun sequencing project [10]. This average coverage was calculated from long stretches of unique sequence, where there was a high confidence in the assembly. This average is half of that of the haploid genome coverage (14), as we are looking at the gene variants in the haploid genome.

### Collapse of coding sequences

Multiple alignments were also built for all annotated genes directly from the assembly. These alignments show which annotated genes are highly similar, by collapsing them. This will give the user information on how many of the estimated copies of a gene already are annotated in the draft genome sequence. The same criteria as for the multiple alignments of shotgun reads were used, i.e. 95% similarity, with the addition that at least 90% of each sequence shorter than the longest annotated gene had to be aligned. This criterion prevents genes with only partial high similarity (e.g. genes that share a similar motif) to be erroneously collapsed.

### Locating repeat cassettes

**Figure 1:**
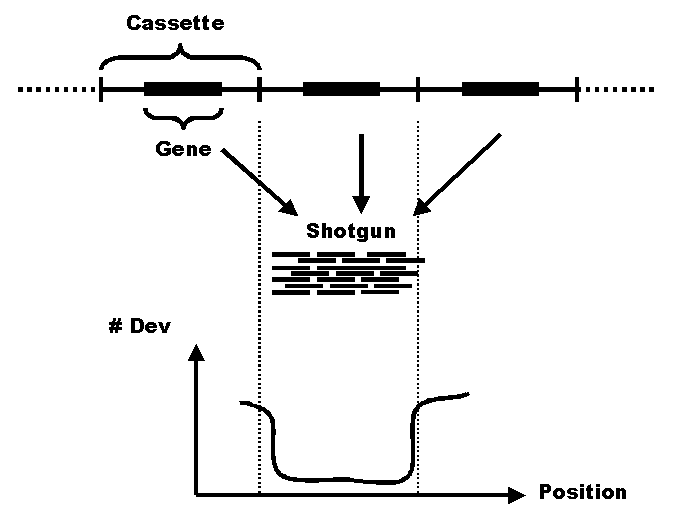
**Cassette estimation**. Schematic description of the procedure for cassette estimation. The top of the figure shows the layout of a repeated region. The repeat unit can consist of a gene, as well as flanking, non-coding sequence, and the repeat array is flanked by unique sequence (indicated by dashed lines). The middle of the figure shows how, typically, the shotgun reads will form a single alignment that represents a merge of all repeat copies. The graph at the bottom of the figure shows the number of deviating bases (y-axis) along the alignment (x-axis). The number of bases deviating from consensus will typically be low within the alignment, and increase towards the ends due to the presence of shotgun reads partially sampling unique sequence.

For several repetitive genes, it was possible to locate the borders of the repeat units. Repetitive units contain both non-coding and coding sequences. Sometimes more than one gene is repeated together, for example the genes encoding tuzin and amastin. Repeat borders were located as follows. The multiple alignment of each annotated gene with an average alignment depth of 100 or more was searched for steps in the column scores. Column scores were calculated by adding the number of differing bases in a column of aligned bases, during the process of realigning the multiple alignment. A single column with an increased number of deviating bases compared to the surrounding columns indicated a polymorphism, while a step to an increased level of deviating bases in several consecutive columns indicated a border between repeated and unique sequence (Figure 1). Such steps are difficult to find for dispersed repeats as well as repeats in large arrays, where the difference in column score would be small. We searched a maximum of 400 bp on either side of the coding sequences to eliminate very thin alignment ends.

Since the cassette borders are somewhat difficult to locate automatically, we used the allele and the comparison with similar annotations, i.e. the annotations from the collapse, for verification. A similar cassette, within an error-margin of 40 bp, was accepted as confirmation of a border.

### The database

A postgreSQL database containing the information on repetitive *T. cruzi *genes can be accessed via a php interface at [19].

### In-depth analysis

Five repetitive genes in the *T. cruzi *genome were chosen for in-depth analysis. For each gene, GRAT was used to locate sequence reads with 95% or higher similarity to two or three annotated versions of the gene sequence, in order to provide complete alignments of the regions. The annotations were chosen using their sequence similarity and their cassette borders. In some cases, the alignments will thus only describe a sub-family, as in the case of the trans-sialidase. The input read dataset consisted of all reads that passed quality control before the published assembly, regardless of whether they were included in the genome assembly or not. As a result, the datasets consisted of reads included in the published assembly, as well as reads that were discarded from the assembly due to the previously mentioned problems with polymorphisms and repeats.

Reads were assembled using Phrap [22], without the use of Phred quality values and with the – *default_qual *parameter set to 10 in order to ascertain that similar reads were aligned. The assemblies were imported into DNPTrapper [21] where the reads were re-aligned and analyzed for DNP content (Defined Nucleotide Positions [23]) in regions with an average Phred quality of 89% or higher. DNPs are instances of single base differences between repeats appearing in reads, as detected by a previously described statistical method distinguishing them from sequencing errors [23].

In each assembly, reads were manually grouped according to DNP content and mate pair data into repeat groups that were subjected to further analysis and comparison. Consensus sequences of the different repeat groups were obtained by a majority vote in regions of depth three or higher. Consensus sequences and their corresponding protein sequences were aligned using ClustalW in order to pinpoint non-synonymous DNPs.

The impact of amino acid differences on protein function in two of the genes was assessed using the prediction software SIFT [24] on alignments built using PSIBLAST [25].

## Results

The genome of *Trypanosoma cruzi *CL Brener is highly repetitive and polymorphic. This makes assembly and subsequent annotation of the genome difficult, and the current draft sequence is fragmented and lacks many copies of highly repetitive genes. We have studied all protein coding genes and pseudogenes in *T. cruzi *for alignment depth using a sensitive multiple alignment tool to build new alignments representing a gene and all its copies.

A database of all annotated genes in *Trypanosoma cruzi *and their repetitive qualities has been created. It can be searched using a locus id or a GenBank id. The information includes estimation of copy number, collapses between annotated genes and, in many cases, repeat cassette information. A few selected genes have been studied in more detail, using the assembly finishing tool DNPTrapper.

### Characteristics of *Trypanosoma cruzi *genes

There are 23 216 predicted protein coding genes and pseudogenes in the published release of the *Trypanosoma cruzi *CL Brener genome. Of these 23 216 sequences, 12 642 (54%), were annotated as hypothetical, and 3 603 (16%) as pseudogenes. Alignments of 22 640 annotated genes were built. This is 98% of all annotated genes. The information gathered in these alignments is stored in the database. Sequences that did not create an alignment are too short or too repetitive for our alignment criteria, or represent possible misassemblies. Too short in this case means shorter than the shortest overlapping read and too long, or too repetitive, means that the resulting alignment was too large to keep in the memory of the computer. Most annotated genes that do not form useful alignments are long and repetitive, with the majority being surface antigens. These will be added to the database at a later date.

There are a few hypothetical genes that did not form alignments, as well as the DnaJ homolog subfamily A member 2 and one version of eukaryotic translation initiation factor 1A. The former was too short to have an overlap with a read and the latter had multiple insertions in the sequence, which made it non-homologous to all of the reads. The insertions might be the result of misassembly of the gene. The lack of overlapping reads for these two genes imply that they are not repetitive.

**Figure 2:**
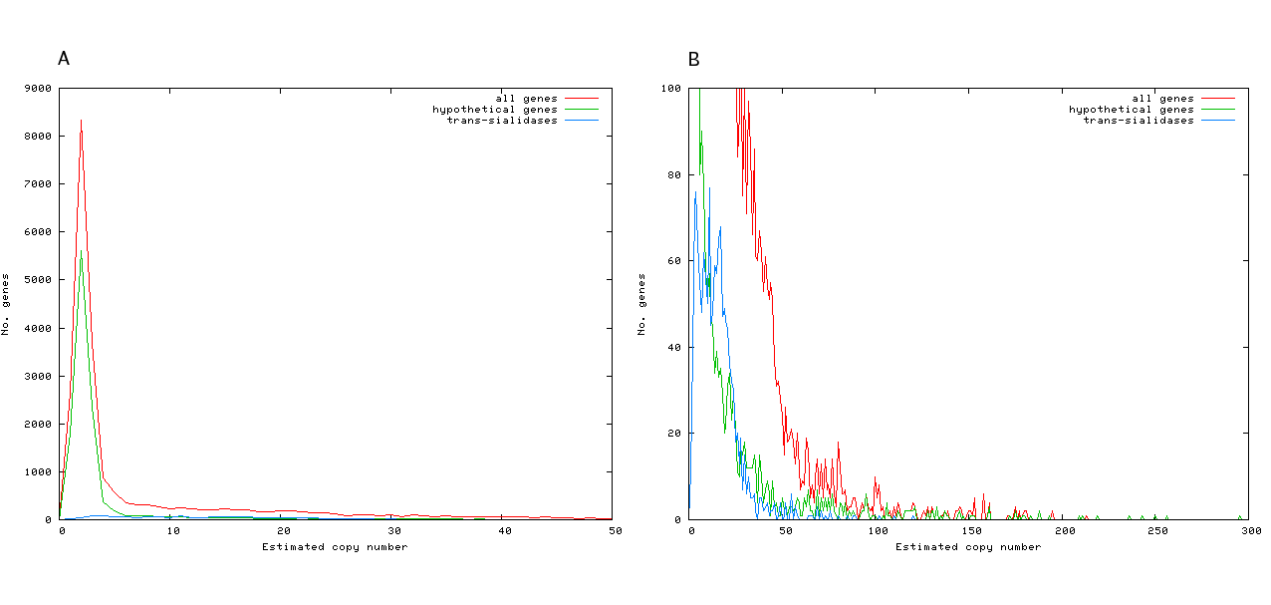
**Distribution of estimated copy numbers**. The estimated copy number is calculated for each annotation by averaging the alignment depth along the annotation and dividing the average by 7, the average shotgun coverage. The distributions of all annotations (red), of all hypothetical genes (green) and of all trans-sialidase annotations (blue) are shown. The distribution shows the number of annotations (y-axis) for each estimate (x-axis). The graph to the left (A) shows the peak of the two first distributions at 2. The graph to the left is a zoomed in version of the higher estimates. The average estimated copy number of the trans-sialidases is 16.

Of the alignments created, 4 171 (18%) showed an average alignment depth of 100 or more. With an average coverage in the sequencing of 7 [10], an average alignment depth of 100 indicates 14 copies in the diploid genome. These 4 171 annotations represent 73 different functional annotations, with mucin-associated surface protein (MASP) being the most common with 913 copies in the published genome with an average coverage over 100. The distribution of the estimates is shown in Figure 2. The distribution for hypothetical genes is shown separately. The figure shows the number of annotations for each copy number estimate. As expected, most annotations have an estimate of 2, where the distribution peaks. The hypothetical genes have a lower fraction of repeated annotations. At a cutoff of 14 copies, which is the cutoff we have used for repetitive genes, the fraction of repeated hypothetical genes is less than a third of that of all annotations (6.6% versus 19.1%). This is clearly visible in Figure 2B, where the distribution of the higher estimates is zoomed in.

A list of the repeated annotated genes is shown in Table 1. This table does not include the hypothetical genes, as they could not be clustered by name. The names are all unique, which means that there could be variants of the annotation that were not included, depending on differences in how the annotations have been curated. This is particularly important for generic gene names, such as for example kinase. Annotations with more specific names are more reliable. The table also shows the number of annotations in the genome assembly that have an average alignment depth of 100 or more (corresponding to an estimated copy number of 14 or above, column 3) and the average estimated copy number of these annotated copies (column 4).

**Table 1:** Draft genome copy numbers and estimated copy numbers of repeated genes

Annotation	No. Genome copies	No. Genome copies w. depth ≥ 100 reads	Avg. No. Estimated copies
trans-sialidase	1430	725	24
MASP	1377	913	23
RHS	752	166	26
TcMUCII	728	708	38
DGF-1	565	231	57
GP63	425	158	27
protein kinase	311	38	36
elongation factor 1-gamma	178	13	18
ATPase	119	5	119
ATP-dependent DEAD/H RNA helicase	99	51	28
UDP-Gal or UDP-GlcNac-dependent glycosyltransferase	66	7	21
beta galactofuranosyl glycosyltransferase	66	52	27
TcMUC	59	44	44
N-acetyltransferase complex ARD1 subunit	58	52	28
TcMUCI	57	55	41
serine/threonine protein kinase	47	6	31
glycine dehydrogenase	37	36	27
mucin-like glycoprotein	28	9	23
receptor-type adenylate cyclase	25	2	17
tryptophanyl-tRNA synthetase	23	21	44
amino acid transporter	22	7	38
histone H4	21	21	79
casein kinase	20	12	123
histone H2A	18	15	100
DnaJ chaperone protein	18	4	23
heat shock protein 70	18	3	100
metallopeptidase	17	4	19
expression site-associated gene (ESAG-like)	16	7	16
cation transporter	16	6	55
myosine heavy chain	13	1	19
mannosyl-oligosaccharide 1, 2-alpha mannosidase 1B	13	9	20
tyrosine aminotransferase	13	9	38
cystathionine beta-synthase	12	12	63
elongation factor 1-alpha	12	10	39
amastin	12	6	19
TcSMUGS	11	11	39
zinc finger protein	11	1	16
histone H3	11	9	42
tuzin	10	8	39
DNA-directed RNA polymerase subunit	10	7	14
cysteine peptidase	9	12	59
helicase	8	1	18
flagellar calcium-binding protein	8	8	66
TcSMUGL	8	8	61
glutamamyl carboxypeptidase	7	6	144
hexose transporter	7	6	35
clathrin coat assembly protein AP19	7	2	76
metacaspase	6	4	25
elongation factor 2	6	3	15
chaperonin HSP60, mitochondrial precursor	5	5	32
calcium-binding protein	5	3	74
folate/pteridine transporter	5	5	20
heat shock protein 85	5	5	54
serine carboxypeptidase (CBP1)	5	5	31
cruzipain precursor	5	4	81
kinase	4	1	32
histone H2B	4	2	63
tricohyaline	4	1	16
D-isomer specific 2-hydroxyacid dehydrogenase protein	4	3	29
membrane-bound acid phosphates	3	1	20
antigenic protein	3	1	18
prostaglandin F2-alpha synthase	3	3	28
mitochondrial RNA editing lipase 1	3	1	15
mitotubule-associated protein Gb4	3	1	15
oligosaccharyl transferase subunit	3	1	19
ubiquitine	3	1	17
69 kDa paraflagellar rod protein	2	2	16
clathrin assembly protein	2	2	76
glucose transporter	2	1	27
pteridine reductase	2	1	18
L-threonine 3-dehydrogenase	2	2	24
TcMUCIII	2	2	38
U3 small nuclear ribonucloprotein snRNP	2	2	25
acetylornithine deacetylase	1	1	153
cytochrome oxidase assembly protein	1	1	16
heat shock protein 83	1	1	60
monoglyceride lipase	1	1	49
paraxonemal rod protein PAR2	1	1	15

The table lists the 78 annotated genes with an average alignment depth of 100 or higher. The table does not include the hypothetical genes. The first column lists the name of the annotated gene. The second column lists the number of copies of that particular annotation in the draft genome. The third column lists the number of these annotated copies that have an average shotgun depth of 100 or higher, corresponding to an estimated copy number of 14 or higher. The fourth column lists the average estimated copy number of those annotated copies having an average shotgun depth of 100 or higher (listed in column three).

As an example, consider flagellar calcium binding protein (FCB). There are eight copies of FCB in the draft genome (column 2). All of these have alignments with an average shotgun depth of 100 or higher (column 3). The copy number estimate of these eight alignments, i.e. the alignment depth divided by the average coverage of the genome sequencing, is, on average, 66 (column 4). In the collapse analysis, each of the genomic copies were grouped with the other seven other copies. In two cases one more annotated gene was in the same collapse (annotated as calcium-binding), and one case two more annotated genes were grouped with the FCBs. These results show that, for FCB, the estimated copy number (66) is the correct total number of FCB genes in the genome.

We chose a stringent cutoff for the average alignment depth (100) in order to ensure that we with high fidelity picked repetitive genes, rather than regions of high shotgun coverage due to random sampling depth. Using the average coverage of the genome sequencing (7×), an average alignment depth of 100 gives an estimated copy number of 14. Interestingly, the estimation of the number of repeat copies for surface antigens and other highly represented annotated genes from our analysis (column 3 in Table 1) was almost always low compared to the total number of family members present in the assembly (column 2 in Table 1). Conversely, high estimates of copy number almost always correspond to annotated genes with few copies in the assembly. This indicates that gene repeats such as the surface antigens, which are driven towards diversity, were in many cases separated in the assembly; whereas highly similar repeats pose a larger problem for the assembly program.

**Figure 3:**
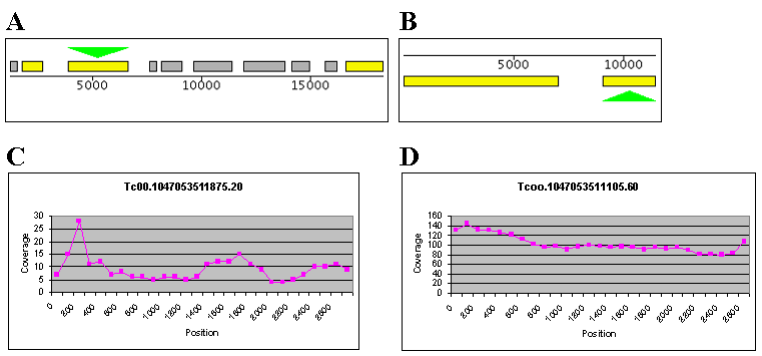
**Coverage of two trans-sialidases**. A, B shows two contigs with annotated putative trans-sialidases. C, D show the coverage every 100 bp along the genes. Tc00.1047053511875 (A, C) has an average shotgun depth of 9, indicating only one copy in the genome. Tc00.1047053511105.60 has an average depth of 103, indicating 15 copies actually being present in the genome. This example shows how trans-sialidases in *T. cruzi *can be both unique (Tc00.1047053511875.20), sequence similarity wise, or closely resemble many others (Tc00.1047053511105.60).

An example is given in Figure 2, where the distribution of the copy number estimations of the 1 430 trans-sialidase annotated copies is shown together with the distributions for all annotations and all hypothetical genes. The average copy number estimation of trans-sialidase in this dataset is 16. If, on the other hand, only alignments with a shotgun depth of 100 or greater are considered, as in Table 1, the average copy number estimate of trans-sialidase is 24. To further clarify, consider the two trans-sialidases in Figure 3. The figure shows the contigs containing the genes and graphs of the alignment depth sampled along the genes. One of the examples has a high alignment depth, while the other appears to only be present as one copy in the genome. This is representative of the trans-sialidases, who, as many of the surface antigen genes, are both divergent enough to be separated in the draft genome and to not be clustered in our analysis, and in some cases similar enough to not be assembled correctly and cluster in relatively large alignments. The estimated copy numbers, represented in column 3 in Table 1, are in cases such as the trans-sialidases estimates of the number of gene variant sub-groups within the larger gene family. In an effort to deduce the actual number of trans-sialidases in the genome, we calculated the average coverage of all trans-sialidase genes. A total of 65 815 reads, with a total length of 47 507 179 bp, formed an alignment with one or more of the trans-sialidases. The trans-sialidases have a total length of 5 783 417 bp in the assembly, which gives an average coverage of 8.2. This is close to the assembly coverage of 7, which indicates that, while there are some collapsed copies, the majority of the trans-sialidase genes were assembled separately. However, since it is clear that many reads aligned to more than one annotated copy it is likely that there are many assembly errors in members of this family, especially at the consensus sequence level, due to regions of high similarity. As a contrast, consider again FCB, which had eight copies in the draft genome and an estimated copy number of 66.

The functional classification of the repeated annotated genes is shown in Table 2. 738 (18%) of the repeated annotated genes were annotated as hypothetical proteins, a somewhat lower fraction than of the entire set. 61% of the annotated genes are surface antigens. The most striking feature in this table is the wide range of functions covered by the repeated genes. Not only surface antigen genes and housekeeping genes, but close to all functional categories have genes that are predicted to be repeated.

**Table 2:** Functional classification of repeated genes

Function	No. of diff. annotations
Metabolism	16
Cell growth, division and DNA synthesis	11
Protein synthesis	4
Protein destination	13
Transport proteins	8
Signal transduction	4
Cellular organization	4
Surface antigens	11
Other	8

The 78 annotations from Table 1 were clustered into functional groups. Note the remarkable spread of function of the repeated genes in *T. cruzi*.

Of the 4 171 alignments with an average alignment depth over 100 that were searched for cassette boundaries, 3 788 (91%) yielded estimates. Of the 1 787 cassettes where the collapse could be used for verification, the borders were supported in 1 318 (74%) cases. Of the 190 cassettes that could be verified using alleles, 118 (62%) were supported. The multiple alignments that did not yield cassette estimations may be dispersed gene repeats or have a larger cassette than our parameters allowed (400 bp on each side).

16 149 annotated genes collapsed with other annotated genes. The remaining sequences either collapsed into one copy in the genome assembly, exist in only one copy, or have two alleles that are too diverse to collapse.

In an attempt to estimate the total gene number of *Trypanosoma cruzi *CL Brener, the total number of basepairs of reads aligned to annotated genes (765 951 reads, 547 513 087 bp) was compared to the total number of basepairs in annotated genes in the original genome assembly (32 883 890 bp). The gene base pair count was corrected for genes annotated as a collapse in the assembly between the two haplotypes (Esmeraldo and non-Esmeraldo). There were 1796 such annotations, and they covered a total of 2 713 442 bp. In total, the annotated genes covered 35 597 332 bp. That gave an average depth over the annotations of 15.4. A comparison with the average coverage in the genome (7) gives an average number of copies per annotation of 2.2. Hence we estimate the number of protein coding genes (including pseudogenes and allelic variants) in *T. cruzi *to be approximately twice the previously estimated amount bringing it up to around 40 000.

### The database

All information gathered in this study is available on-line. The data are stored in a postgreSQL database, which is accessible through a php interface. The data can be searched using a locus id or a GenBank id. The user can also upload a file of locus ids or GenBank ids. At the top of the results page there are links to the respective GeneDB and GenBank pages. GeneDB [26] is a repository of the official annotation and genome browser for *T. cruzi*. An additional database and browser (TcruziDB) can be accessed at [27].

There are four choices of how much information to show for each search: estimated copy number, collapse, COGs, and cassette. The estimated copy number shows the average depth of the multiple alignment, the standard deviation of a sampling of the depth and the estimated copy number of the annotated gene. There is a link, *Details*, to the sampled depth, so that the user can see the distribution. The database can also be searched by contig or by estimated copy number.

The collapse shows the annotated genes that are highly similar to the query (at least 90% of the shorter of the two is in the alignment). A link at the collapsed sequence, *Info*, gives the user information on the putative function, allele, possible split junction, and hybrid strain of the annotation. A link at the end of the collapse information, *Get Seqs*, gives the DNA sequences of all annotated genes in the alignment, including the query.

The COGs information was taken directly from the *T. cruzi *genome project, where the COGs were formed using jaccard clustering [10]. The annotated genes in a COG share potential function, and complement our collapse data.

The cassette data is only available for certain queries. It shows the number of basepairs of cassette sequence before and after the start and end of the annotation. There is also information on whether or not the annotations in the collapse verify the cassette borders and whether or not the other allele verifies them. A link, *Info*, provides additional information on the cassette, such as if the cassette contains more than one annotations.

A list of the reads participating in an alignment can be reached through the link *Get the read list *at the end of the result page. The reads and the quality values are available at the database website.

### In-depth analysis

The genes selected for further analysis highlight different characteristics of *T. cruzi *repetitive genes, with respect to organization, conservation and other features. They also illustrate the types of useful information that can be extracted from the database in combination with the DNPTrapper software.

### Allelic tandem arrays

Tyrosine aminotransferase (TAT), codes for an aromatic amino acid transferase, similar to the corresponding tyrosine catabolism enzymes found in rat and human liver [28]. For *T. cruzi*, TAT has been annotated in nine contigs in GeneDB, in most cases at the end of a contig or in a small contig by itself, indicating that the assembler used for the whole genome assembly of *T. cruzi *(Celera assembler, [29]) was not able to extend the contigs in the TAT regions due to repeats.

We used two of the 13 annotated gene variants to search for reads. These two sequences were in the same COG, collapsed together, and both indicated a copy number for this particular version of TAT of 47 in the gene repeat analysis described above. Using GRAT, 1 357 reads matching the region within the similarity threshold were retrieved. Out of these, 710 were not included in the assembly and were thus analyzed for the first time in the present study.

Using DNP Trapper, the reads could be divided into two large groups of roughly the same size (group 1: 670 reads with maximum alignment depth 210, indicating approximately 30 copies; group 2: 560 reads, depth 205, around 30 copies) based on their DNP content. Analysis of the distribution of mate pairs from shotgun clones supported this division in that there were many mate pairs within both groups and none between the groups. This supports the hypothesis that TAT is present in two allelic tandem arrays in the genome, one on each of a homologous pair of chromosomes. Furthermore, this illustrates a previously observed phenomenon in *T. cruzi*, where repetitive genes differ in sequence between homologs but are more conserved within homologs [10,21]. In the intergenic region, however, the DNP patterns were less homogenous in both groups, allowing for further division into around 20 distinct subgroups for group 1 and 10 subgroups for group 2. 120 reads could not be reliably assigned to either of the two large groups, because of lack of, or ambiguous, DNP information.

The DNPs in the coding region were investigated to see if they represent differences in the corresponding amino acid sequences. In group 1, three subgroups with DNPs in coding positions were identified. Group 2 had two such subgroups, one of which had an alignment depth of 140, indicating that the majority (20 out of 30) copies in this group had sequence that was divergent from that available in the public databases.

The amino acid changes were analyzed for possible functional effects using the prediction software SIFT. We created an alignment using PSI-Blast, with median sequence conservation at the prediction sites of 2.62, except at the first amino acid change where it was 3.09. All SNPs were predicted to be tolerated except the first, which was a Valine to Tyrosine change and had a SIFT score of 0.00. It had, however, a low 'Seq Rep' value (0.41), which indicates a poor prediction [24].

Similar results were obtained for flagellar calcium binding protein (FCB), which is believed to be important for trypanosomatid flagellar mobility [30], and has been proposed to be used in serological diagnosis of Chagas' disease due to the *T. cruzi *specificity of the C-terminus [31].

There are 8 annotations of FCB in three contigs in GeneDB, with all contigs breaking at the FCB site. In two of these, FCB is flanked downstream by hypothetical proteins that are homologous to each other, while the third contig consists of two merged copies. In the read retrieval, we used two of these annotated variants of the gene as well as one gene annotated as calcium-binding protein, which collapsed with the two FCBs but was not in the same COG, as queries. Two of these annotated gene variants had an estimated copy number of 73, the remaining one an estimation of 72. 1 100 reads matching the region were located, of which 869 were not included in the genome assembly.

The reads could again be divided into two large groups of 540 and 470 reads, respectively, based on DNP content, also in this case supported by mate pair data. Approximately 220 reads could not be assigned to any group. Again, this indicates that the gene is present in allelic tandem arrays on both homologs. From the maximum alignment depth in each group (470 and 400 respectively), the copy number was estimated to be 70 copies in group 1 and 60 in group 2.

Six DNPs in the coding regions were found in coding positions, one of which was present in 160 reads or around 20 copies in one of the groups. We performed a SIFT analysis on the amino acid changes as above. The median sequence conservation was between 3.06 and 4.32, with a majority of 3.06. No amino acid changes were predicted to be deleterious.

### Extremely conserved repetitive gene

*T. cruzi *heat shock protein 85 (HSP85) has homology to hsp90 in *S. cerevisiae*, hsp83 in *D. melanogaster*, and hsp90 in chicken, and has been proposed to be a target of the autoimmune response in parasite infection [32]. It has been annotated in three contigs in GeneDB, all ending in tandem copies of the gene (in one of the cases the contig actually ends in the highly similar HSP83).

We used three of the five HSP85 gene annotations as queries. The three sequences collapsed together and were in the same COG (as previously determined by clustering in [10]). They all had an estimated copy number of 63. 444 reads matching the region were present in the assembly, and an additional 1279 were retrieved by GRAT, giving a total of 1 723 reads. Unlike the previous two regions, this data set could not be readily divided into two groups. This was mainly due to the lack of DNPs in the coding region: 75% of the reads do not have reliable DNPs in this region at all. The gene copies are thus so similar, even between alleles, that they cannot be separated using these methods.

Analysis showed that the few DNPs present in the coding regions are at wobble positions (except one identified case with a frame shift, probably a pseudogene), which indicates that HSP85 shows a high degree of conservation in *T. cruzi*.

### Highly repeated surface antigen gene

Trans-sialidase transfers sialic-acid residues from host glycoconjugates to parasite mucins. It can also bind to mammalian cell receptors and undermine host defense mechanisms [33]. The protein has an enzymatic domain on the N-terminus that is required for trans-sialidase activity. In the C-terminus, there are varying numbers of 12 amino acid antigenic repeats. There is limited divergence in the amino acid sequence of the N-terminal and there is one amino acid in particular (Tyr342) that is required for enzymatic activity [34]. Most experiments that involve trans-sialidase only take the N-terminal, enzymatic region into account [35,36].

As mentioned above, 1 430 trans-sialidase genes have been annotated in the *T. cruzi *CL Brener genome sequence, twelve of which have been found to contain the critical Tyr342. Inactive versions have previously been found to have a histidine in this position [34], but none of the inactive annotated variants in the current version of the genome contain this histidine.

As an example of how these types of gene families can be studied using our methods, we used two of the 1 430 annotated variants of trans-sialidase to search for reads. The two sequences were in the same collapse and COG, both had an estimated copy number of ten and both were predicted to have trans-sialidase activity.

**Figure 4:**
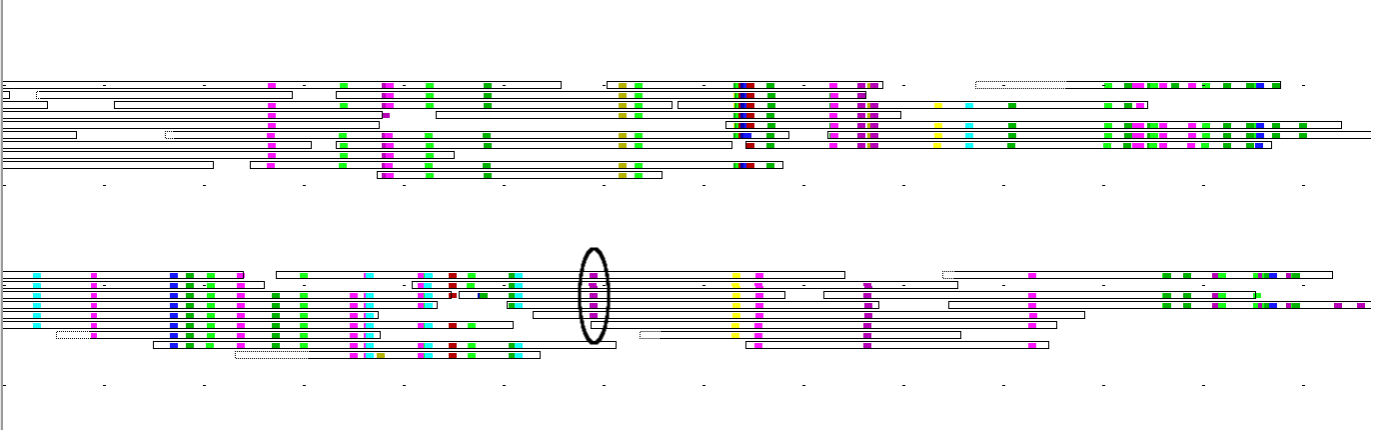
**Active and inactive copies of trans-sialidase**. Comparison of two trans-sialidase repeat groups in DNPTrapper. Boxes indicate reads, colored dots indicate DNPs. Only part of the alignment is shown. The reads have been clustered in DNPTrapper based on their DNP content, with reads sharing similar DNP patterns being grouped together. The lower group contains a C – T base substitution (circled) that corresponds to a Tyr – His substitution in the protein, rendering this repeat copy to lose its trans-sialidase activity.

The DNA sequences corresponding to the relatively conserved N-terminus of the active annotations were used for retrieving matching reads, resulting in 516 reads within the similarity threshold of 95%. 185 of these were not present in the assembly. Nine read groups covering most of the region were chosen for further analysis, while a few groups were discarded due to low coverage, ambiguous DNPs and in one case a frame shift indicating a pseudogene. Furthermore, three additional groups were identified as inactive in the critical position (Figure 4), while still having strong similarity to the region. The fact that only three inactive copies out of 1 418 were found indicates that once the gene copies have lost their trans-sialidase activity, they have diverged rapidly.

Out of the nine groups, four matched annotated gene variants perfectly, while the additional five groups showed similarities of 93% to 98% to annotated copies. The inactive groups had similarities between 90% and 97% to the annotated copies.

### Repetitive gene with differences in predicted transmembrane regions

There are 11 812 sequences from *T. cruzi *in GeneDB that have been annotated as coding for hypothetical proteins. We chose one of these for further analysis. The corresponding protein is expressed in epimastigotes of *T. cruzi*, as shown by performing mass spectrometry on a sub cellular fraction of the parasite (Ferella, unpublished). The protein has several predicted transmembrane regions and contains a Nodulin like motif (PF06813). Proteins containing this Pfam signature are for example the peptide transporter PTRZ in *S. cerevisiae*, the organic anion transporting peptides (OATPs) in human and rat, and the BT1 family, such as the pteridine transporters found in *L. major*. All these are transporters with similar numbers of transmembrane domains, and it is therefore possible that the *T. cruzi *counterpart is involved in transport.

In the assembly, this hypothetical protein was part of a seven-member COG. We used two annotated variants that collapsed together and had similar cassette boundaries. The two sequences had estimated copy numbers of 27 and 28, respectively.

973 reads matching the entire gene region were extracted. The distribution of these reads was found to be somewhat uneven. The alignment depth indicated that the first ~80% of the gene has a copy number of around 60, whereas the last 20% showed lower copy numbers, around 40. 467 of the reads were not included in the genome assembly.

**Figure 5:**
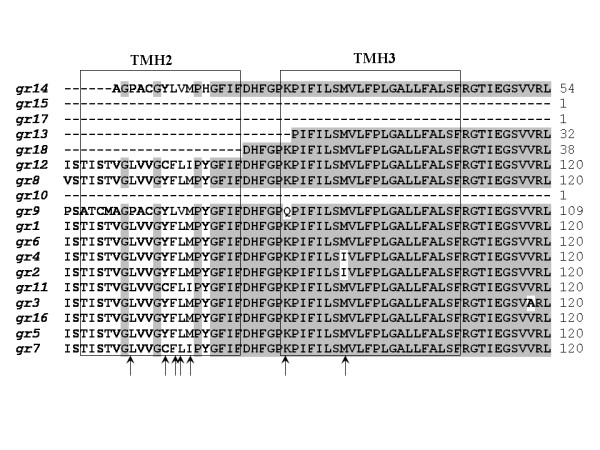
**Protein sequence alignments of gene with transmembrane regions**. Protein sequence alignment of 17 good coverage groups, from EAN81429.1. The names of the amino acid sequences to the left represent the read group their consensus sequence was derived from. Boxes show a region of the sequences with two predicted transmembrane helixes (TMH2 and TMH3). The arrows indicate positions inside the TMH regions where there is an amino acid change. It is worth to notice that most of the differences are seen in TMH2 but not as much in TMH3. Identical residues are shaded. Left numbers show the sequence position.

Out of the 40 DNP groups that could be identified, 17 with good coverage over most of the protein coding sequence were chosen for further investigation. We observed that a relatively large number of single base differences between copies of the gene give rise to amino acid differences between the corresponding proteins. 46 such positions were identified, not considering the C-terminal region where most of the copies differ in sequence as well as in length. 35 of these changes are located in transmembrane regions (identified using Phobius [37]), and although most of them are favored substitutions, they could possibly give rise to different substrate specificities (Figure 5).

## Discussion

Although the *T. cruzi *genome may be regarded as unusually repetitive, repeated DNA is present in all genomes, with repeat unit lengths ranging from a single nucleotide to several mega bases. In this study, we have focused on the phenomenon of repetitive genes, where the same protein coding sequence, or variants thereof, is present in multiple copies in a genome.

Understanding of the processes that give rise to repeats is necessary in order to better understand fundamental mechanisms involved in evolution, biology and disease. Through the sequencing of the human and other higher eukaryote genomes in recent years, new light has been shed on instances where repeats are involved. Large duplications that in turn consist of smaller-sized "duplicons" present in several locations in the human genome provide a modularity that makes different compounds and complex gene combinations possible [38,39]. One consequence of repetitive genes is a diversification of protein function, where Hox genes are one example among many [40]. Short repeats are involved in chromatin remodeling [41], and recent concepts such as gene factories [42] and noise control [43] include gene repeats as key elements. Repetitive genes are also implicated in different diseases, such as cancer [44,45], developmental and neurological disorders [1], and others [46]. Conversely, gene repeats also play a role in plant disease resistance [47].

This exhaustive study of all predicted protein coding sequences and pseudogenes in *T. cruzi *illuminates the overwhelmingly repetitive and complex nature of this genome. Not only the surface antigens are repeated, but also genes involved in a variety of functions from metabolism to cellular organization. Several genes with only one annotated copy in the genome were found to be present in dozens of copies. The copy number estimates reported here are based on sequence coverage and are in most cases reliable. It is possible that there are some slight overestimates caused by uneven read distribution. This can easily be verified by the variance of the alignment depth or by viewing the distribution of the sampled depth directly. The estimate of the total number of genes in *T. cruzi *has potential sources of error. The reads that align to an annotation do not always align fully, and there can be some partial overlaps at the edges of the annotation. This is, however, a minor error-source, since the annotations in general are several times longer than a read length, and since the depth of the alignments always decreases rapidly outside of the annotation. The second error source is the trimming of the reads. We have quality trimmed the reads to a minimum of 97%, which yielded an average read length of 704 bp. The reads in the assembly have an average read length of 681. This is also a minor (3%) difference, and should not influence the results significantly. A third potential error-source is the average coverage used to estimate the copy numbers. This average coverage was taken directly from the genome paper and is expected to be accurate. An average coverage of 7.5 instead of 7 would reduce an estimate of 50 to 47 and an average coverage of 6.5 would add four copies, resulting in an estimate of 54. The fact remains that the *T. cruzi *genome is far more repetitive in terms of gene content than previously reported. We have, in this study, counted the two allelic copies of a gene as two gene variants, making our estimate of 40 000 gene variants in *T. cruzi *a haploid count in a diploid genome. This is, however, how the original gene count, assembly and annotation was made due to the hybrid nature of the sequenced strain. The previous estimate is about half of what we have discovered here.

The genome annotation is based only on contigs longer than 5 kb or contigs belonging to scaffolds longer than 5 kb of the genome assembly. Many more contigs were assembled, but considered too fragmented for genome annotation. This fragmentation is likely due to the repeated nature of *T. cruzi*, and it is possible that some of the novel gene variants we report here in fact are located on these un-annotated contigs. Since they have not been annotated, they are not included in current gene databases, gene counts or the presented assembled sequences; the database we present in this study is an attempt to rectify this.

The analysis has been performed on the genome sequencing reference strain, CL Brener. It is likely that other strains have different gene copy numbers. CL Brener has a relatively large genome when compared to other strains, which can in part be explained by the higher degree of repeated sequence [48]. CL Brener is, however, the strain sequenced and the information provided here is a complement to its draft genome sequence.

In *T. cruzi*, different functions have been suggested for the repetitive genes, such as evasion of the host immune system [10] and increased expression in the absence of promoters [49]. It is clear from our genome-wide analysis and in-depth study of a few selected genes that *T. cruzi *has a wide range of gene repeats with different characteristics. Tyrosine aminotransferase and flagellar calcium-binding protein are examples of genes present in tandem arrays on each homolog, with a high degree of conservation within homologs and more differences between homologs. Although there are differences between repeat units, they rarely cause amino acid changes and when they do, they are in most cases not likely to induce changes in protein function. HSP85 is an example of an extremely conserved gene with virtually no differences between repeat copies, indicating that there is great selective pressure to keep this gene intact. Trans-sialidase exemplifies the highly repetitive surface antigens, and has another interesting characteristic: seemingly, the loss of an amino acid crucial to trans-sialidase activity reduces the pressure to keep the gene intact and allows it to rapidly evolve into highly divergent variants. Finally, the hypothetical protein included in the analysis is an example of a gene that exists in many versions, with amino acid differences likely to alter protein function. We show here that our database combined with DNPTrapper makes it possible to efficiently study all these different gene families in detail.

## Conclusion

Our study shows that the genome of *T. cruzi *contains far more information than previously reported. In our estimate, approximately twice the amount of genes predicted from the genome assembly is actually present in the genome. This includes a correction for merged haplotypes, where the two allelic copies of a gene have been assembled into one. This has implications for future studies of *T. cruzi *genetics and biology. Further investigation of the repetitive genes may provide important insight into the biology of the parasite, as well as better understanding of gene repeats at a general level. Furthermore, before initiating analysis of particular genes, it is crucial to find out whether they are repeated or not, as this may influence the outcome of PCR, cloning and functional studies. For instance, the hypothetical protein and trans-sialidase included in our in-depth study clearly exist in many different versions, and it is important to realize that the versions found in GeneDB may not even be present in the genome. Our database makes it possible to carry out thorough bioinformatics analyses of regions of interest before experiments are designed, and thereby minimize the risk of making uninformed assumptions that influence the final results.

The task of carrying out a genome wide repeat analysis is straightforward using the methods described here, and the result enables scientists to look further into repeated regions of interest with more accurate information at hand. None of the programs used in this study (GRAT, DNPTrapper, ClustalW, SIFT and Phobius) are time consuming, or difficult to use. We predict that this type of analysis will be an important complement to future genome projects, and suggest that similar endeavors can be carried out also for other already sequenced genomes. The presence of repeats is still a major problem in shotgun sequencing, and with a possible shift from Sanger sequencing towards high throughput methods generating short reads without mate pairs, the repeat problem will continue to cause errors in shotgun assemblies. Future assembly algorithms may incorporate improved methods to deal with repetitive elements, but in the current situation, we feel it is highly questionable whether it is cost-effective to try to produce completely finished sequence within the framework of an individual genome project. The Assembly Archive in combination with databases such as the one presented here, together with the software we have used, will enable scientists specifically interested in different regions to finish those parts themselves, making the finishing of genomes a public effort similar to the Open Source process in computing.

## Authors' contributions

EA performed the in-depth analysis, helped plan the project and draft the manuscript. EK built and analyzed the alignments, created the database, helped plan the project and draft the manuscript. DN and MTT helped plan the project. FF and MF helped perform the in-depth analysis. BA helped plan the project and draft the manuscript. All authors read and approved the final manuscript.
